# Interaction of two imidazolium gemini surfactants with two model proteins BSA and HEWL

**DOI:** 10.1007/s00396-015-3671-z

**Published:** 2015-07-08

**Authors:** W. Gospodarczyk, M. Kozak

**Affiliations:** Department of Macromolecular Physics, Faculty of Physics, Adam Mickiewicz University, ul. Umultowska 85, PL61614 Poznan, Poland

**Keywords:** Gemini surfactants, BSA, HEWL, Protein aggregation

## Abstract

Gemini surfactants and their interactions with proteins have gained considerable scientific interest, especially when amyloidogenic proteins are taken into account. In this work, the influence of two selected dicationic (gemini) surfactants (3,3′-[1,8-(2,7-dioxaoctane)]bis(1-dodecylimidazolium) chloride and 3,3′-[1,12-(2,11-dioxadodecane)]bis(1-dodecylimidazolium) chloride) on two model proteins, bovine serum albumin (BSA) and hen egg white lysozyme (HEWL), have been investigated. A pronounced and sophisticated influence on BSA structure has been revealed, including a considerable change of protein radius of gyration as well as substantial alteration of its secondary structure. Radius of gyration has been found to rise significantly with addition of surfactants and to fall down for high surfactants concentration. Similarly, a remarkable fall of secondary structure (α-helix content) has been observed, followed by its partial retrieval for high surfactants concentration. A strong aggregation of BSA has been observed for a confined range of surfactants concentrations as well. In case of HEWL-gemini system, on the other hand, the protein-surfactant interaction was found to be weak. Molecular mechanisms explaining such behaviour of protein-surfactant systems have been proposed. The differences of properties of both studied surfactants have also been discussed.

## Introduction

Protein-surfactant systems have potential applications in wide range of industrial aspects, including the following: drug delivery, cosmetics, food industry and preparation of pharmaceutical substances, as well as in biotechnology and biosciences [1–3]. Proteins are often components of healthcare products because they have affinity to bind various molecules as well as catalyse biochemical reactions (for example, superoxide dismutase) [4]. Proteins are known to attach diverse surfactant molecules, giving protein-surfactant complexes, where hydrophobic parts of surfactant molecules tend to bind interior hydrophobic residues of proteins [5]. Studying these phenomena can help in understanding the effect of surfactants on protein denaturation, solubilisation and renaturation processes [6, 7]. A very important phenomenon is also protein aggregation, which can be responsible for many serious human diseases (including number of neurodegenerative diseases) and it is often regarded as undesired effect in biotechnology [8–10]. Its molecular mechanism is still not entirely deciphered and poses challenge [11].

There are relatively few scientific reports concerning the interaction of proteins with a novel class of dicationic amphiphilic compounds, called gemini surfactants. These surfactants consist of two polar groups and two hydrophobic chains. They exhibit higher surface activity, better solubility and capability of foaming in comparison to conventional (monomeric) surfactants [12, 13]. A variety of structural forms of gemini surfactants allows their properties adjustment dependently on the length of hydrophobic chains and the polar groups distance, as well as their overall chemical structure. Usually, much lower concentrations of gemini surfactants are required to perform desired function, which implies their limited impact on environment [14]. They form in water the structures similar to the structure of biological membrane, which can additionally diminish possible toxicity on human body in medical applications [15]. These surfactants are a very interesting group of surfactants due to their other unusual, in comparison to their conventional surfactant homologs, properties [16, 17].

Bovine serum albumin (BSA) is a well-studied transport protein often used as a model system [4, 18]. Serum albumins are one of the most abundant proteins present in many organisms blood plasma [19]. Bovine serum albumin has molecular mass about 66 kDa, consists of 583 amino acids [20] and is the homolog of human serum albumin. This protein comprises three domains and is stabilised by 17 disulphide bridges [21, 22]. It was found to possess binding sites for many different substances, e.g. aromatic ligands, fatty acids and metals [23]. Another protein often used as a model system is hen egg white lysozyme (HEWL). It is a rather small protein composed of 129 amino acids [24, 25], with molecular mass of 14.4 kDa, having two domains, stabilised by four disulphide bridges [26]. Hen egg white lysozyme is hydrophobic residues rich and is highly positively charged at neutral pH [14, 25]. HEWL is also one of the most known model proteins suitable for aggregation processes examination, as its molecular structure and physicochemical properties are well established [27, 28]. Both BSA and HEWL are appropriate proteins for mechanisms of proteins-surfactants interaction examination [29]. Whilst several scientific groups have investigated interaction between BSA and various gemini surfactants [30, 31], there are very few studies on interaction of gemini surfactants with lysozyme [14].

**Fig. 1:**
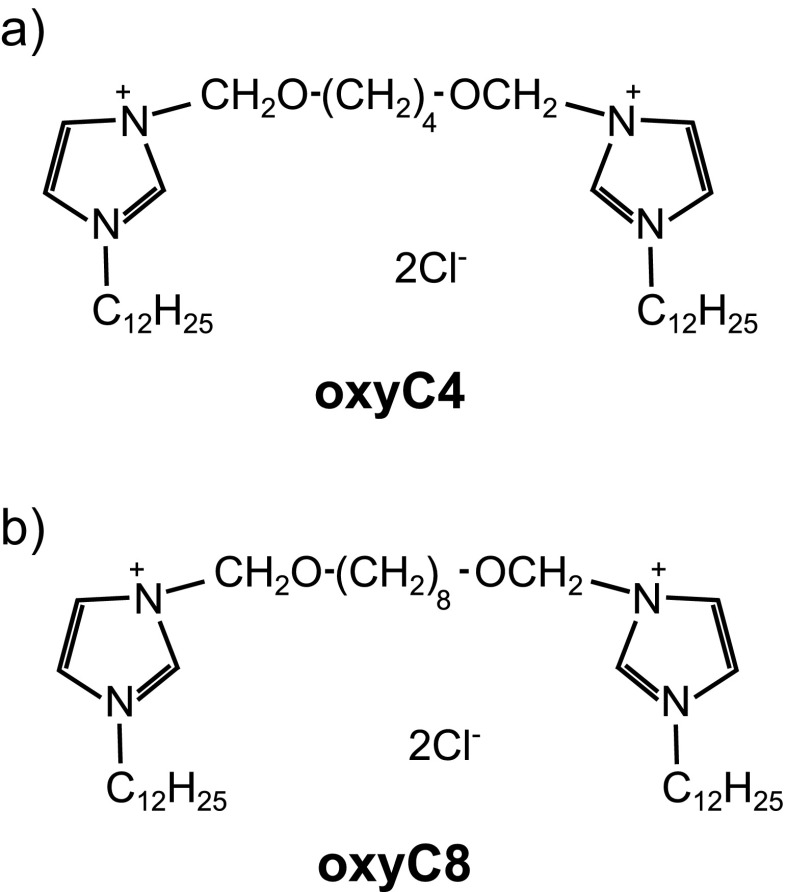
Chemical structure of surfactants used in the study: **a** 3,3′-[1,8-(2,7-dioxaoctane)]bis(1-dodecylimidazolium) chloride (oxyC4), **b** 3,3′-[1,12-(2,11-dioxadodecane)]bis(1-dodecylimidazolium) chloride (oxyC8)

The aim of our work was to find out the characteristics of interactions between model proteins (BSA and HEWL) and two novel gemini surfactants: 3,3′-[1,8-(2,7-dioxaoctane)]bis(1-dodecylimidazolium) chloride (oxyC4) and 3,3′-[1,12-(2,11-dioxadodecane)]bis(1-dodecylimidazolium) chloride (oxyC8) (see Fig. 1 for their chemical structure). We have used two complementary experimental techniques to achieve this aim: circular dichroism (CD) and small angle scattering of synchrotron radiation (SAXS). The former method allows determination of the secondary structure of an examined protein, whereas the latter one provides information on the tertiary structure (in example, the radius of gyration of the protein). However, each technique requires different values of protein concentration; in CD, measurements of proteins concentration were equal to 0.4 mg/ml (BSA) and 0.8 mg/ml (HEWL), whilst in SAXS, the concentration was chosen to be 4 mg/ml (for both proteins). To carry out comparable measurements with the use of both techniques, we set surfactant-to-protein molar ratios similar within both methods.

## Materials and methods

### Samples

Gemini surfactants 3,3′-[1,8-(2,7-dioxaoctane)]bis(1-dodecylimidazolium) chloride (oxyC4) and 3,3′-[1,12-(2,11-dioxadodecane)]bis(1-dodecylimidazolium) chloride (oxyC8) were synthesized in a modified way described earlier [32, 33]. Their chemical structure is presented in Fig. 1. BSA, HEWL and sodium dihydrogen phosphate dihydrate were purchased from Sigma Aldrich.

All samples were prepared as protein-surfactant solutions with use of phosphate buffer solution at pH equal to 7.3 ± 0.4.

### Small angle X-ray scattering

Small angle X-ray scattering (SAXS) measurements were performed on P12 beamline of EMBL Hamburg Outstation on PETRA III storage ring at DESY [34–36]. Scattering data were recorded using a Photon counting Pilatus 2 M pixel detector (253 × 288 mm^2^) at the sample-to-detector distance of 3500 mm. The detector *s*-axis (where *s* = 4πsinθ / λ with 2θ the scattering angle, wavelength *λ* = 0.15 nm) was calibrated using the diffraction patterns of silver behenate [37]. The scattering vector range was 0.08 > s > 4.5 nm^−1^. The measurements were carried out on a series BSA and HEWL samples containing the gemini surfactants at increasing concentrations. The SAXS data were collected in 20 successive 0.1 s frames. All measurements were performed using a capillary cell (sample volume 10 μl) and automated filling at 25 °C. The collected frames were integrated and averaged.

Reference SAXS data sets for 15.2 mM solutions of oxyC4 and oxyC8 surfactants were collected on the BM29 beamline [38] of ESRF (Grenoble, France) using synchrotron radiation (*λ* = 0.9919 nm). The scattering experiments were done at 15 °C with sample-to-detector distance of 2.867 m, using flow cell (sample volume = 30 μl) and Pilatus 1 M detector (169 × 179 mm^2^). For each sample, 10 frames (10 s) were collected.

The scattering data were corrected for detector response, normalised to the incident beam intensity, and the scattering of the buffer was subtracted using the programme package PRIMUS [39]. Solution of a known concentration (~3 mg/mL) of xylose/glucose isomerase from *Streptomyces rubiginosus* was used as reference [40].

To derive information about overall size of proteins, radii of gyration of BSA and HEWL were determined as follows. Guinier relation was used, which state that the natural logarithm of SAXS intensity versus square of scattering vector ln*I*(*s*^2^) is linear for small *s*^2^, and the slope of this dependence is proportional to square of radius of gyration, *R*_g_^2^. ln*I*(*s*^2^) curves were plotted in PRIMUS, and the best linear fit was performed manually, with respect that *sR*_g_ should be greater than 0.1 and should not exceed 1.4. The value of *R*_g_ for a particular fit together with experimental errors was computed by PRIMUS.

### Circular dichroism

Circular dichroism (CD) spectra were collected with use of Jasco J-815 CD Spectrometer at room temperature. The spectral range was 190–260 nm. The measurement scanning speed was equal to 50 nm/min, data was pitched every 0.5 nm and five accumulations were taken for every spectrum. The path length was equal to 0.2 mm.

To derive information about secondary structure of proteins, the spectra were deconvoluted by CDSSTR [41] programme with reference to SP175 database [42], both available at Dichroweb server [43].

We have performed a few additional measurements of solutions transmittance at 250–400 nm. The parameters here were as followings: scanning speed 200 nm/min, data pitch 0.5 nm, 3 accumulations, path length 2 mm (in case of BSA samples of 4 mg/ml) or 5 mm (in case of 0.4 mg/ml BSA samples). Results for 400 nm were taken into account.

## Results and discussion

### SAXS

**Fig. 2:**
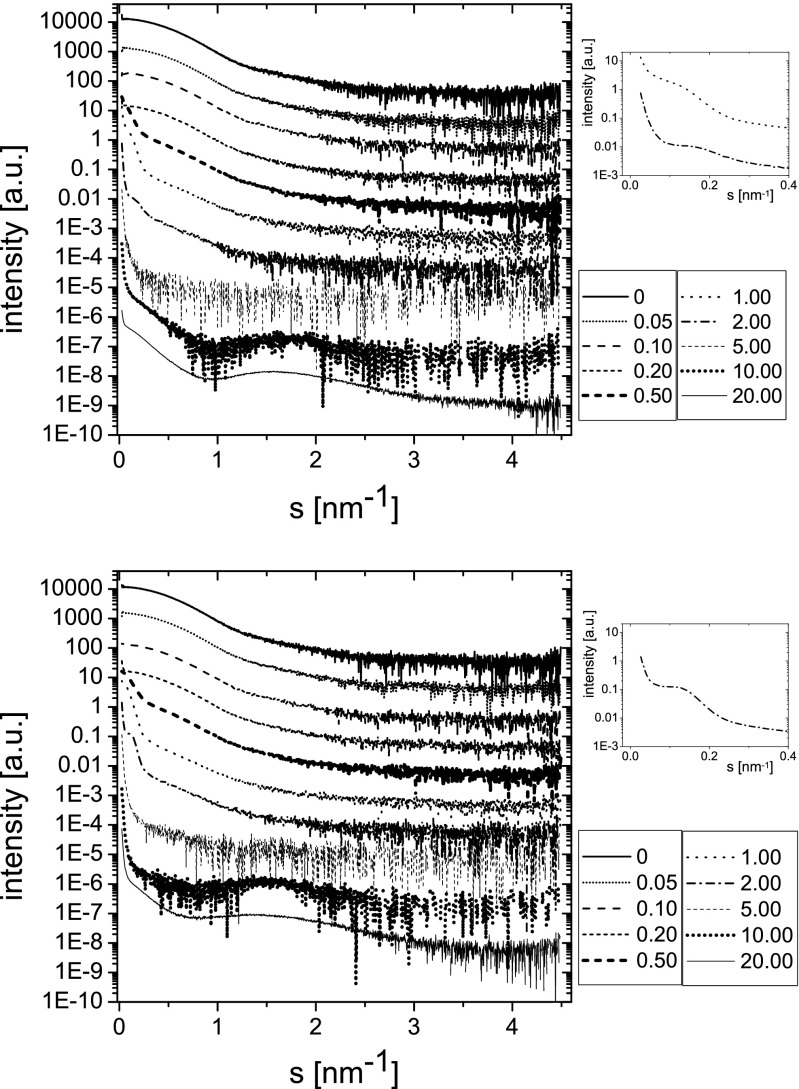
SAXS patterns of BSA/oxyC4 (*top panel*) and BSA/oxyC8 (*bottom panel*) solutions. BSA concentration was 4 mg/ml. The surfactant concentrations in millimolar are given in the panels. Insets—magnification of *s*-range 0–0.4 nm^−1^ for BSA/oxyC4 (surfactant concentration 1 and 2 mM; *top panel*) and for BSA/oxyC8 (surfactant concentration 2 mM; *bottom panel*)

**Fig. 3:**
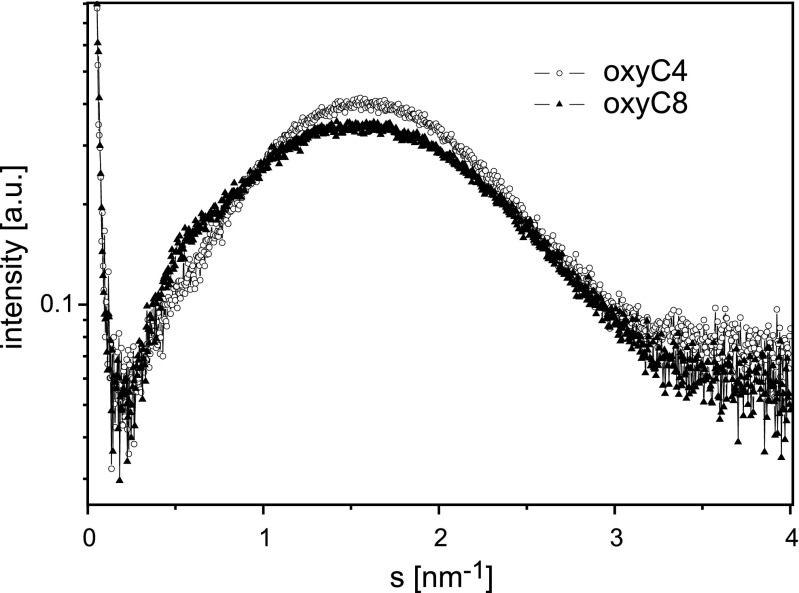
SAXS patterns of oxyC4 and oxyC8 solutions (concentration 15.2 mM)

SAXS curves obtained for BSA/oxyC4 and BSA/oxyC8 solutions for a full range of surfactant concentrations are presented in Fig. 2. Reference SAXS curves recorded for 15.2 mM solutions of oxyC4 and oxyC8 are presented in Fig. 3. Radii of gyration calculated by the fit of experimental SAXS data to Guinier equation are listed in Table 1. At low concentrations of surfactants, the radius of gyration characterising systems study starts to rise at a slow pace. Then, at surfactants concentration of 0.5 mM, a sudden growth of gyration radius is observed, up to about five times the initial *R*_g_. For most of the SAXS curves collected for greater values of *c*_surf_, the radius of gyration *R*_g_ was found to be indeterminable. Most of the samples of *c*_surf_ range of 0.5–10 mM turned turbid to a different extent, and white precipitate occurrence in few of these cases could be also observed. This effect went through its maximum and was weaker at final surfactant concentrations. SAXS curves from this region, especially for *c*_surf_ equal 2.0, 5.0 and 10.0 mM, revealed increased noise level. At the final concentration of 20 mM, the radius of gyration for BSA/oxyC4 solution was found to be determinable and equal to 7.1 nm. At high surfactant concentrations (10 mM, 20 mM), a secondary maximum at SAXS curves is present in the *s*-range of about 1–2 nm^−1^. This broad maximum is associated with the formation of micelles by studied surfactants (see Fig. 3).

**Table 1:** Radii of gyration of BSA as a function of surfactants oxyC4 and oxyC8 concentrations (*c*_surf_) and as a function of the surfactant-to-BSA molar ratios (*c*_surf_:*c*_BSA_)

*c*_surf_ [mM]	0	0.05	0.10	0.20	0.50	1.00	2.00	5.00	10.00	20.00
*c*_surf_:*c*_BSA_	0	0.8	1.7	3.3	8.3	17	33	83	170	330
*R*_g_ [nm] (BSA/oxyC4)	2.99 ± 0.01	3.02 ± 0.01	3.05 ± 0.01	3.17 ± 0.01	16.5 ± 0.2	–	–	–	–	7.1 ± 0.1
*R*_g_ [nm] (BSA/oxyC8)	2.99 ± 0.01	3.02 ± 0.01	3.06 ± 0.01	3.28 ± 0.01	12.8 ± 0.1	17.7 ± 0.1	–	–	–	–

“–” symbol indicates inability of radius of gyration determination for a given solution

**Fig. 4:**
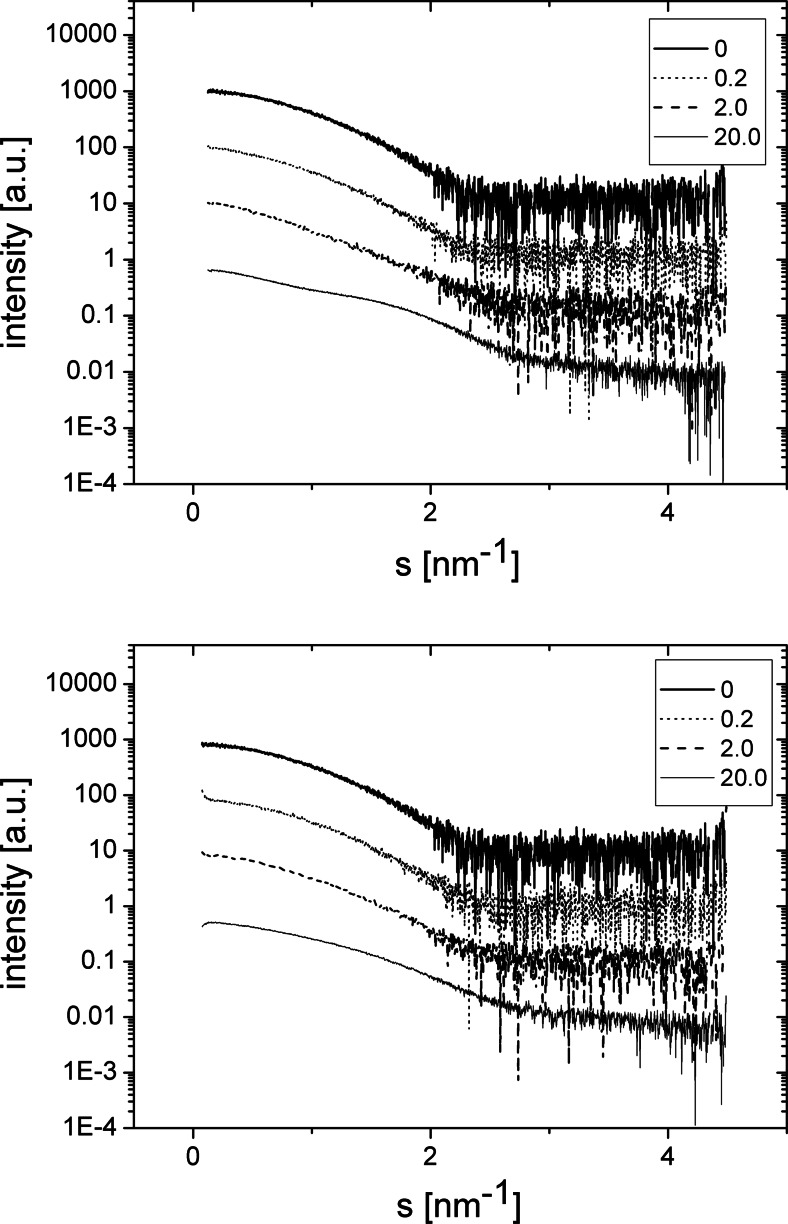
SAXS patterns of HEWL/oxyC4 (*top panel*) and HEWL/oxyC8 (*bottom panel*) solutions. HEWL concentration was 4 mg/ml. The surfactant concentrations in millimolar are given in the panels’ insets

A very different dependence of HEWL molecules on surfactant addition was observed (Fig. 4 and Table 2). Here, the radii of gyration characterising this protein are slightly, but distinctly, increased upon interaction with oxyC4 surfactant. For the second surfactant, the changes are similar but less pronounced, and for HEWL/oxyC8 (20 mM) sample, a decrease of HEWL radius of gyration to 1.5 nm was observed.

**Table 2:** Radii of gyration of HEWL as a function of surfactants oxyC4 and oxyC8 concentrations (*c*_surf_) and as a function of the surfactant-to-HEWL molar ratio (*c*_surf_:*c*_HEWL_)

*c*_surf_ [mM]	0	0.2	2.0	20.0
*c*_surf_:*c*_HEWL_	0	0.7	7.2	72
*R*_g_ [nm] (HEWL/oxyC4)	1.68 ± 0.01	1.82 ± 0.01	1.98 ± 0.01	1.84 ± 0.01
*R*_g_ [nm] (HEWL/oxyC8)	1.68 ± 0.01	1.71 ± 0.01	1.77 ± 0.01	1.49 ± 0.01

### Circular dichroism

**Fig. 5:**
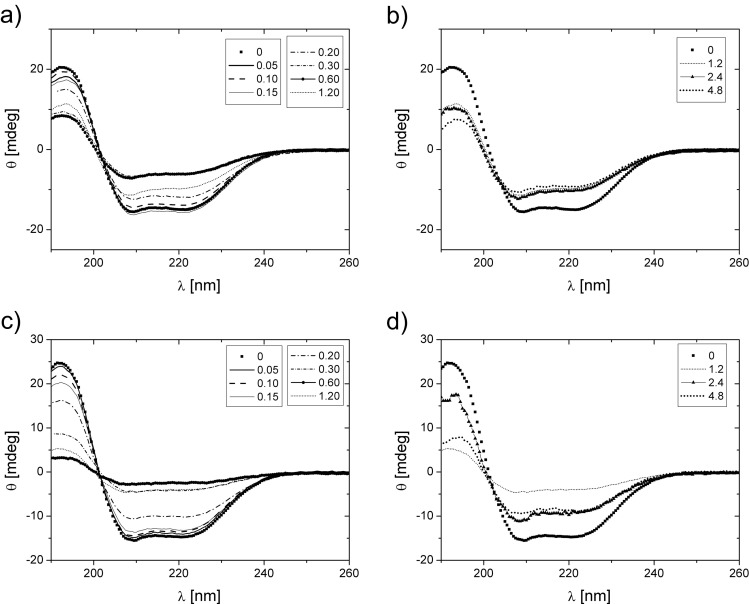
CD spectra obtained for BSA/oxyC4 samples in a low (**a**) and high (**b**) surfactant concentration values region and for BSA/oxyC8 samples in a low (**c**) and high (**d**) surfactant concentration values region. The BSA concentration was 0.4 mg/ml; surfactants concentrations in millimolar are listed in the panels’ insets

CD spectra collected for BSA are presented in Fig. 5. The content of α-helixes in the secondary structure of the examined protein, derived from these spectra, is shown in Table 3. In the first region of surfactant concentrations, α-helicity of BSA is approximately unchanged or slightly decreased. Then, for both surfactants, a rapid transition of BSA secondary structure is observed to a form containing substantially less α-helixes. For higher surfactant concentrations, BSA exhibited a partial restoration of α-helix content, which was observed to take place more easily in case of oxyC4 surfactant. Spectrum of BSA/oxyC8 (0.6 mM) sample was not of sufficient quality to resolve the secondary structure of the protein. Some samples of intermediate concentrations of surfactants were found to turn slightly turbid. This effect, however, was not as much pronounced as in the case of SAXS samples, due to significantly smaller amounts of interacting molecules present in these solutions. Here, again, the effect was found to go through a maximum and paled afterwards.

**Table 3:** α-helicity of BSA protein as a function of oxyC4 and oxyC8 surfactants concentrations (*c*_surf_) and as a function of the surfactant-to-protein molar ratio (*c*_surf_:*c*_BSA_)

*c*_surf_ [mM]	0	0.05	0.10	0.15	0.20	0.30	0.60	1.20	2.40	4.80
*c*_surf_:*c*_BSA_	0	8.3	17	25	33	50	100	199	399	797
α-helicity [%] (BSA/oxyC4)	62	61	57	65	52	25	29	47	48	46
α-helicity [%] (BSA/oxyC8)	60	56	56	53	44	18	–	17	43	42

“–” symbol indicates inability of determination of α-helicity for a given solution

**Fig. 6:**
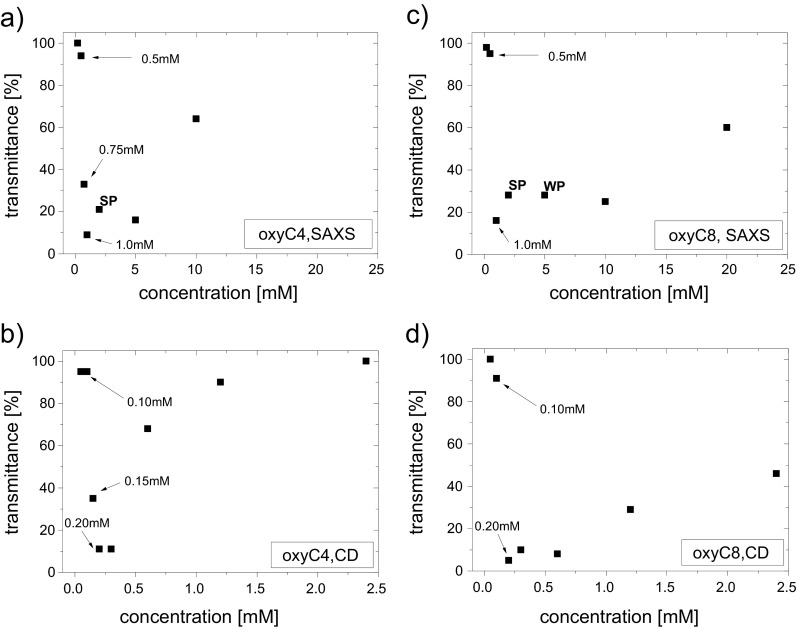
Transmittance of majority of surfactants-protein solutions in the study, with reference to transmittance of BSA(4 mg/ml)/surfactant(0 mM) for SAXS solutions and BSA(0.4 mg/ml)/surfactant(0 mM) for CD solutions, as a function of surfactant concentrations, measured for **a** oxyC4 SAXS samples, **b** oxyC4 CD samples, **c** oxyC8 SAXS samples, and **d** oxyC8 CD samples. In these experiments, the optical path length was 2 mm for SAXS samples or 5 mm for CD samples. Plots for a given surfactant are displayed in such a way that the molar surfactant-to-BSA ratios range is the same in respective figures. SP and WP stand for cases of strong or weak precipitate deposition, respectively

To examine the effect of decreased transparency of solutions in a quantitative, rather than qualitative manner, we have measured optical density of most of the samples used in SAXS and CD experiments to reveal the dependence of solution turbidity on surfactant concentration. These results are presented in Fig. 6. All curves exhibit a very steep downfall of transmittance of the solution which is entirely due to turbidity of solutions. In case of BSA(4 mg/ml)/oxyC8(2 mM), the extent of the “turbidity” effect was stronger than in case of, in particular, BSA(4 mg/ml)/oxyC8(1 mM) sample, including a strong precipitate deposition, but the solution itself was found more transparent for light as a consequence of decreased amount of material in solution apart from precipitate. A weaker precipitate was also observed in case of BSA(4 mg/ml)/oxyC8(5 mM) sample and a rather intense for BSA(4 mg/ml)/oxyC4(2 mM) sample.

**Fig. 7:**
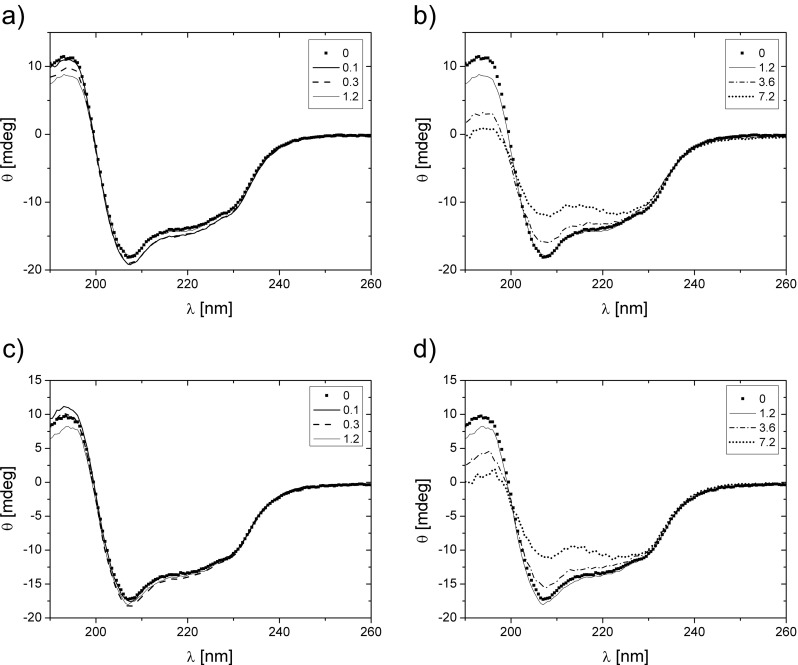
CD spectra obtained for HEWL/oxyC4 samples in a low (**a**) and high (**b**) surfactant concentration values region and for HEWL/oxyC8 samples in a low (**c**) and high (**d**) surfactant concentration values region. The HEWL concentration was 0.8 mg/ml; surfactants concentrations in millimolar are listed in the panels’ insets

CD spectra measured for HEWL and calculated secondary structure are presented in Fig. 7 and Table 4, respectively. For a wide range of concentrations of both surfactants (oxyC4 and oxyC8), the protein does not exhibit any visible change of the content of α-helixes, up to about 1.2 mM, and is equal to approximately 38 %. Then, it decreases gradually to 23–24 % at surfactant concentration of 7.2 mM.

**Table 4:** α-helicity of HEWL protein as a function of oxyC4 and oxyC8 surfactants concentrations (*c*_surf_) and as a function of the surfactant-to-protein molar ratio (*c*_surf_:*c*_HEWL_)

*c*_surf_ [mM]	0	0.1	0.3	1.2	3.6	7.2
*c*_surf_:*c*_HEWL_	0	1.8	5.4	22	65	129
α-helicity (%) (HEWL/oxyC4)	38	38	39	38	36	24
α-helicity (%) (HEWL/oxyC8)	37	37	39	38	35	23

### BSA and gemini—low surfactants concentration regions

At the first stage of interaction, the secondary structure of BSA is preserved. The α-helical content is unchanged and the radius of gyration slowly increases. At small concentrations, gemini surfactants were found to stabilise the structure of BSA, by attaching their hydrophilic heads to negatively charged residues present at the surface of BSA [44, 45], which exhibits negative net charge at pH 7.7 (its isoelectric point is equal to about 4.5) [46, 47]. Hydrophobic alkyl chains of surfactant molecules bind with hydrophobic residues present at the protein surface [44].

After gemini surfactants concentration reached 0.5 mM, a rapid growth of protein *R*_g_ was observed. Indeed, at the second stage of interaction, surfactants hydrophobic tails are expected to enter the interior of BSA leading to unfolding of the protein and therefore increasing protein radius of gyration [7]. Another process influencing radius of gyration occurs when more and more surfactant molecules bind to the protein. The negative net charge of BSA diminishes upon attaching many positive gemini molecules, and then the protein lacks solubility [48]. This effect is clearly visible in examined solutions. Decreased net charge stops preventing protein aggregation; therefore, this process is an independent factor affecting *R*_g_. A very pronounced and sudden increase of radius of gyration of BSA for both gemini surfactants may presumably be, to some extent, driven by both of these factors. However, according to the subsequent analysis, the main factor seems to be the protein neutralization.

### Importance of molar surfactant-to-BSA ratio

In the case of strongly negatively charged BSA, the protein can effectively bind positively charged molecules and the concentration of free gemini molecules in solution can be quite low, especially at low and moderate surfactant concentrations. Thus, it seems that it is the molar surfactant-to-BSA ratio that is largely responsible for the system properties, more than absolute values of surfactant concentrations. In both prepared SAXS and CD samples, BSA could be neutralised around the molar ratio of gemini-to-BSA of 8.3:1, rather than e.g. 17:1, as the charge of gemini surfactants is equal to 2, and BSA net charge at pH of 7 is −17 [48]. As pointed above, reduction of protein net charge results in molecules attraction, and at *c*_surf_:*c*_BSA_ = 8.3, a significant growth of BSA *R*_g_ was observed.

The relevance of the assumption about importance of protein-to-surfactant molar ratio can be reinforced when results of solutions transparency measurements are taken into account (Fig. 6). Here, a sudden downfall of system transmittance, which is to be attributed to similar state of compared systems, occurs at similar molar ratios in both samples used for SAXS and CD experiments. The character of changes of transmittance is also very much alike.

**Fig. 8:**
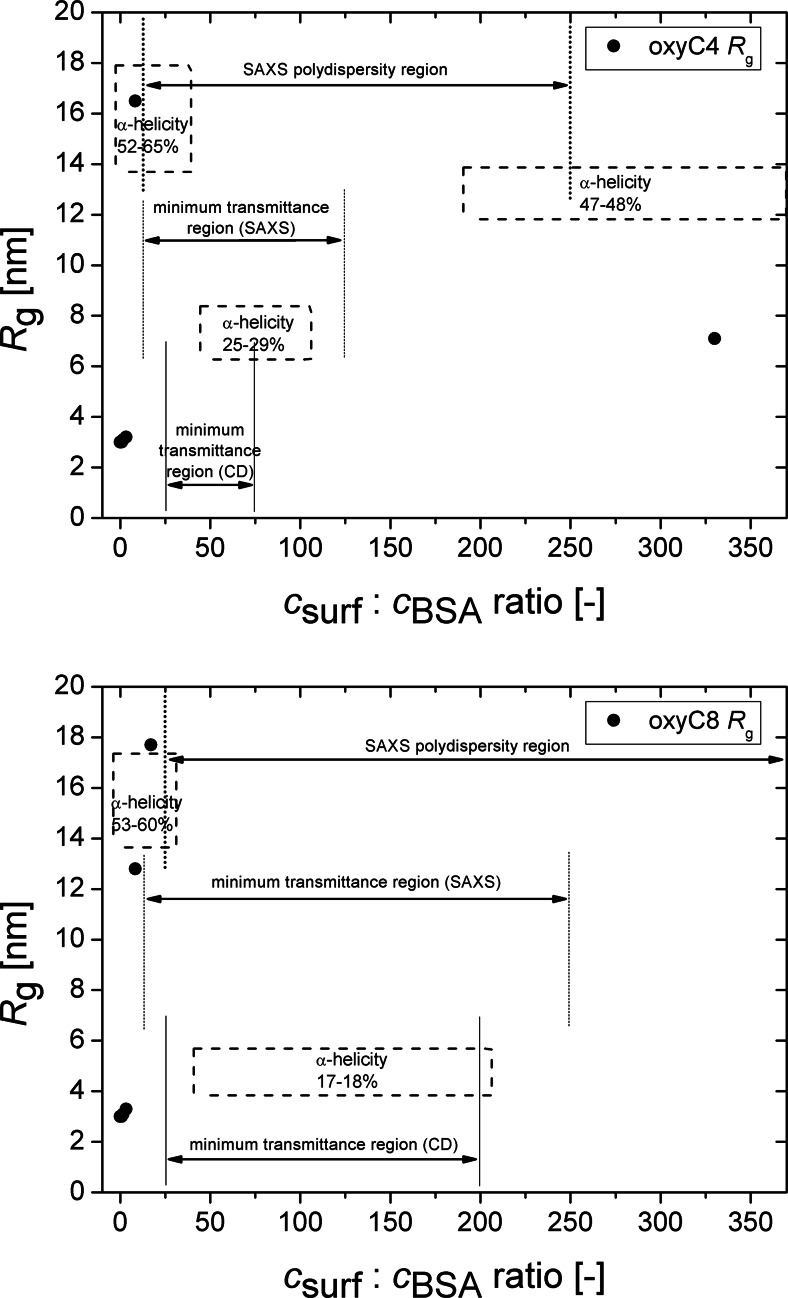
Dependencies of BSA radius of gyration on the molar surfactant-to-protein ratio for both studied gemini surfactants: oxyC4 (*top panel*) and oxyC8 (*bottom panel*). *Vertical lines* indicate the regions of plots where the clarity of BSA/surfactant solutions was poor or where radii of gyration could not be determined, accordingly. *Dash-line boxes* show dependence of BSA α-helicity on molar surfactant-to-protein ratio

To help compare data from different figures and tables, Fig. 8 sets together main BSA/oxyC4 and BSA/oxyC8 systems state indicators: radius of gyration, α-helicity, low transmittance region and inability of *R*_g_ determination (denoted as “SAXS polydispersity region”), in dependence on surfactant-to-BSA molar ratio. Analysing this graph, the molar surfactant-to-BSA ratio seems to be the major, but not the only, factor governing system properties.

### Interaction between gemini surfactants and BSA

Comparison of data presented in Fig. 6 with data from Tables 1 and 3 makes an important contribution to understanding the BSA-gemini surfactant interaction. Firstly, SAXS dependencies clearly reveal that the abrupt rise of *R*_g_ of BSA takes place prior to transparency loss of sample solution (Fig. 6 and Table 1). The second process comes right after the former one. Secondly, dependencies recorded during circular dichroism studies show that the pronounced downfall of α-helix content occurs well within the region of transmittance minimum (Fig. 6 and Table 3). Upon binding of dicationic surfactant molecules, therefore, BSA first increases its radii of gyration, about the region of significantly reduced net charge; soon afterwards, the system considerably loses solubility and undergoes aggregation, with protein secondary structure only partly disturbed. Then, at the moment of the strongest aggregation (with marked precipitate deposition), rapid transition of BSA to low-α-helix state occurs. Taken these facts into account, the rapid growth of radius of gyration should be attributed to protein aggregation driven by weakening of BSA net charge. As a result, aggregates are formed, still not big enough to disrupt solution monodispersity. Subsequently, the impact of gemini surfactants on BSA secondary structure results in protein unfolding, preceded by even stronger aggregation. After *R*_g_ rapid rise, when strong aggregation occurs, the radii of gyration cannot be found. As Guinier relation, underlying procedure of *R*_g_ determination is based on assumption of solution comprising only of monodisperse particles; the samples in this range are very likely polydisperse.

Analyses about system state in higher concentrations of gemini surfactants may have a more speculative character. Aggregation persists for a range of surfactant concentrations before it relents (what can be evidently seen from solution transparency recovery), even despite the increased BSA positive net charge resulting from binding more molecules of dicationic surfactant. BSA unfolding process could promote aggregation state if protein segments were to bind with segments of other molecules. Low-α-helix state coexists with solution turbidity and system polydispersity; the transition to partial restoration of α-helix content takes place shortly before solutions complete clarification (Figs. 6b, d and 8). In the final stage of interaction, when aggregation is weakened, BSA partially renaturates to a more structuralized form (with increased α-helix content). On the other hand, however, the above-mentioned aggregation persistence could as well be due to weak attraction between BSA and molecules of gemini surfactants at this stage of system interactions. The conclusions derived from analysis on the difference between examined surfactants, discussed below, could favour the former option.

### Differences between surfactants in study

The influence exerted on BSA by oxyC4 surfactant is somehow different than the influence of oxyC8 surfactant. In SAXS experiment, the state of the system polydispersity persisted to greater concentrations for oxyC8 surfactant (Table 1). In CD measurements, oxyC4 reduced the BSA α-helix content to a smaller extent and the low-α-helicity state lasted over a shorter range of surfactant concentration (Table 3). Transmittance measurements show that turbidity occurring in solutions is removed by addition of smaller amounts of oxyC4 than oxyC8 gemini molecules (Fig. 6). However, the starting points for each of the processes: rapid *R*_g_ growth, abrupt α-helix downfall and sharp loss of solutions transparency, take place at particular surfactant concentrations, regardless of whether these phenomena are triggered by oxyC4 or oxyC8 surfactant. Summing up, surfactant oxyC8 causes a stronger and more long-term unfolding and maintains aggregation state more efficiently. This fact, combined with partial coexistence of the low-α-helicity state and aggregation state, could suggest that unfolding is a factor distinctly contributing to aggregation persistence in a region where BSA regains electrical net charge.

The length of spacer group is the only factor that differs the molecules of oxyC4 and oxyC8 and must therefore be solely responsible for differences in interaction with BSA. For surfactant oxyC8 spacer is four methylene groups longer, which could promote surfactant increased flexibility, enabling easier entering into the interior of BSA molecule. Additionally, oxygen atoms, which modify the spacer polarity, are more separated for oxyC8 molecule, which could also be a factor promoting protein unfolding.

### Micellar activity of surfactants

Analysing the system properties, it is also vital to consider micellar activity of studied surfactants. The critical micellization concentration (CMC) value for oxyC4 and oxyC8 surfactants is 0.51 mM and 0.71 mM, respectively [49]. If the majority of surfactant molecules put into solutions bind, until *c*_surf_:*c*_BSA_ of order of 8.3, with BSA, the effective concentration of free molecules is much lower than nominal. Therefore, solutions of both dicationic surfactants with BSA will not contain micelles until concentrations which are higher than CMC, e.g. about 1.0 mM and 1.2 mM for oxyC4 and oxyC8, respectively. In case of higher *c*_surf_ values, BSA aggregates and micelles could coexist, but, however, any sign of micellar activity is not observed in SAXS until samples of *c*_surf_ equal 10 mM and 20 mM (Fig. 2); curve for BSA(4 mg/ml)/oxyC8 (5 mM) might exhibit the same characteristic pattern but a very slight one. Micelle formation is supposed to be disturbed by interaction with aggregating protein molecules; after neutralisation of BSA surface charge, gemini surfactant molecules could further bind to BSA. Surfactant tails not only can bind to hydrophobic residues of unfolded BSA, but they can also attach to tails of gemini molecules already bound with BSA. Therefore, they can form micelle-like structures bound to (or surrounding) the protein molecule. Not until this process saturates do the free micelles appear in the solution. This mechanism has been described by Li and co-workers and Ge and co-workers [44, 45].

In CD samples, on the other hand, BSA surface charge can be neutralised in much lower values of surfactant concentrations (of order of 0.05 mM), far less than CMC values. As a result, the solution could contain free micelles at concentrations of surfactants as low as 0.56 mM for oxyC4 and 0.76 mM for oxyC8. Such differences of micellar properties between respective samples (of the same molar surfactant-to-BSA ratio) in SAXS and CD experiments should be taken into account when comparing data obtained with use of these two techniques. Exemplarily, for a sample of molar surfactant-to-BSA ratio of 50 SAXS samples could already contain free micelles, whereas in CD samples, any free surfactant molecules are not to assemble to any form of micelles.

For SAXS curves of BSA/oxyC4(2 mM) and BSA/oxyC8(2 mM) a marked peak is visible, at small *s* values of 0.10–0.20 nm^−1^ for oxyC4 and 0.07–0.25 nm^−1^ for oxyC8 (shown in an inset of Fig. 2), which can be attributed to particles with size of tens of nanometers. The peak is much more subtle but observable, also for BSA/oxyC4(1 mM) sample. Li and co-workers and Ge and co-workers [44, 45] proposed that at high gemini surfactant concentrations, the structure of BSA-gemini complex could be necklace-like, as micelles formed by surfactant may bind to unfolded chain of protein. A structure similar to this could even be formed with gemini micelles built in the structure of folded protein. The above-mentioned peaks could therefore take origin from these structures or other, similar, formed in the solution. This analysis proves that at high surfactant concentrations, the complex structure may be sophisticated.

### HEWL and gemini surfactant interactions

The HEWL-gemini surfactant interactions are presumably mainly driven by electrostatic and hydrophobic interaction. Both HEWL macromolecule as well as surfactant molecules are positively charged (HEWL pI is about 11 [50, 51]) at pH of 7.7, which diminishes the capability of binding with each other. Amiri et al. [14] found that interaction of lysozyme with gemini surfactants was substantially weaker at pH of 4 or 7 than at pH of 11. However, positively charged surfactant heads can attach to non-distantly located negatively charged amino acid residues, including Asp and Glu residues present in HEWL sequence [52], whilst surfactant aliphatic tails can bind to hydrophobic regions of protein surface [14, 53]. Positively charged morpholinium surfactants were reported [20] to bind to lysozyme aromatic residues and make this protein undergo similar change of hydrodynamic radius (as found in fluorescence correlation spectroscopy experiment) to the change in radius of gyration revealed in our SAXS experiment. This group observed protein radius increase by about 25 %, followed by its decrease with increasing surfactant concentration. In our case, the radius was increased from 1.7 to 2.0 nm and 1.8 nm for surfactants oxyC4 and oxyC8, respectively (Table 2). After this growth, the radius was finally found to be 1.9 nm in case of oxyC4 and 1.5 nm in case of oxyC8. On the other hand, positively charged arginine molecules was also found to bind to positively charged lysozyme molecule and induce significant reduction of the hydrodynamic radius of lysozyme [20]. In our study, results for surfactant oxyC8 (Table 2) reveal loss of HEWL radius of gyration of about 10 % compared to its radius in protein native state.

The secondary structure of HEWL is preserved over a quite broad range of dicationic surfactant concentrations (Table 4). Summers et al. [53] observed similar effect when they investigated the influence of ethylammonium nitrate salt on lysozyme. It was suggested that salt’s cation bound to positively charged lysozyme molecule with its hydrophobic group and—at the same time—cation’s positive charge stabilised lysozyme secondary structure. This might be the mechanism of oxyC4 and oxyC8 attaching to HEWL, which was found responsible for interaction of lysozyme with other positively charged molecules as well. For example, Wang et al. [54] reported that 1-butyl-3-methylimidazolium chloride cation, a positively charged molecule with a short (butyl) hydrophobic tail, bound to lysozyme by attaching its hydrophobic region to the protein molecule. In addition, Banerjee et al. [55] found that cation of tetraethylammonium bromide, a small, positively charged molecule, also interacted with lysozyme mainly by its hydrophobic groups.

Circular dichroism spectroscopy (Table 4) showed lack of change of HEWL secondary structure over a wide range of surfactant concentrations, whereas SAXS results (Table 2) revealed small but distinct disturbance of tertiary structure. Morpholinium salts exhibited similar impact on lysozyme, such as the disturbed protein tertiary structure, with much weaker disturbance of protein secondary structure, especially in the region of small or moderate surfactant concentrations [20].

We did not observe any aggregation, including precipitate formation, in HEWL samples. Such aggregation is mainly driven by intermolecular hydrophobic interaction of protein molecules [53]. For example, ethylammonium nitrate salt’s cationic group was found to inhibit lysozyme aggregation by binding to hydrophobic sites of the protein, therefore, preventing lysozyme hydrophobic sites to bind with sites of other protein molecules [53]. Such mechanism could well be responsible for lack of aggregation in HEWL/oxyC4 and HEWL/oxyC8 solutions.

At high concentrations of dicationic surfactants, a pronounced decrease of HEWL α-helix content is observed (Table 4). Investigation of ethylammonium nitrate salt on denatured lysozyme revealed that this salt promoted protein folding, but at higher surfactant concentrations, however, it induced lysozyme denaturation [53]. On the other hand, at small salt concentration, its impact on protein structure was insignificant [53]. High concentrations of morpholinium salts favoured unfolded state of protein and stabilised denatured lysozyme [20]. In the light of these considerations, the evident modifications in HEWL α-helix content at high values of gemini surfactant concentrations may therefore be a start of HEWL denaturation process, where protein unfolds and losses secondary structure characteristic for its native state.

## Conclusions

The study revealed a strong impact of both gemini surfactants on BSA molecule. This influence can be summarised as an initial protein stabilisation, followed by a rapid and pronounced protein radius of gyration increase and loss of solution transparency and monodispersity, then a sudden downfall of α-helix content (partial protein unfolding) and finally, transparency retrieval (solution of micelles and BSA or BSA-micelle complexes) and partial retrieval of initial α-helix content. In the case of HEWL-gemini systems, the surfactant-protein interaction was rather weak leading to moderate modifications of protein radius of gyration and lysozyme secondary structure. There have been found differences in effect exerted on proteins by the two gemini surfactants: oxyC4 and oxyC8 and in case of BSA-gemini systems, it has been observed that surfactant oxyC8 induced more pronounced and longer-lived changes of BSA structure.
